# Mosquito proboscis-inspired needle insertion to reduce tissue deformation and organ displacement

**DOI:** 10.1038/s41598-020-68596-w

**Published:** 2020-07-22

**Authors:** Annie D. R. Li, Ketut B. Putra, Lei Chen, Jeffrey S. Montgomery, Albert Shih

**Affiliations:** 1 Department of Mechanical Engineering, University of Michigan, Ann Arbor, MI USA; 2 Department of Biomedical Engineering, University of Michigan, Ann Arbor, MI USA; 3 Department of Urology, University of Michigan, Ann Arbor, MI USA; 4 Department of Psychiatry, University of Michigan, Ann Arbor, MI USA

**Keywords:** Mechanical engineering, Biomedical engineering

## Abstract

This study investigates mosquito proboscis-inspired (MPI) insertion applied to the clinically used biopsy needle to reduce tissue deformation and organ displacement. Advanced medical imagining has enabled early-stage identification of cancerous lesions that require needle biopsy for minimally invasive tissue sampling and pathological analysis. Accurate cancer diagnosis depends on the accuracy of needle deployment to the targeted cancerous lesion site. However, currently available needle delivery systems deform and move soft tissue and organs, leading to a non-diagnostic biopsy or undersampling of the target. Two features inspired by the mosquito proboscis were adopted for MPI insertion in prostate biopsy: (1) the harpoon-shape notches at the needle tip and (2) reciprocating needle-cannula motions for incremental insertion. The local tissue deformation and global prostate displacement during the MPI vs. traditional direct insertions were quantified by optically tracking the displacement of particle-embedded tissue-mimicking phantoms. Results show that the MPI needle insertion reduced both local tissue deformation and global prostate displacement because of the opposite needle-cannula motions and notches which stabilized and reduced the tissue deformation during insertion. Findings provide proof of concept for MPI insertion in the clinical biopsy procedures as well as insights of needle–tissue interaction for future biopsy technology development.

## Introduction

**Figure 1:**
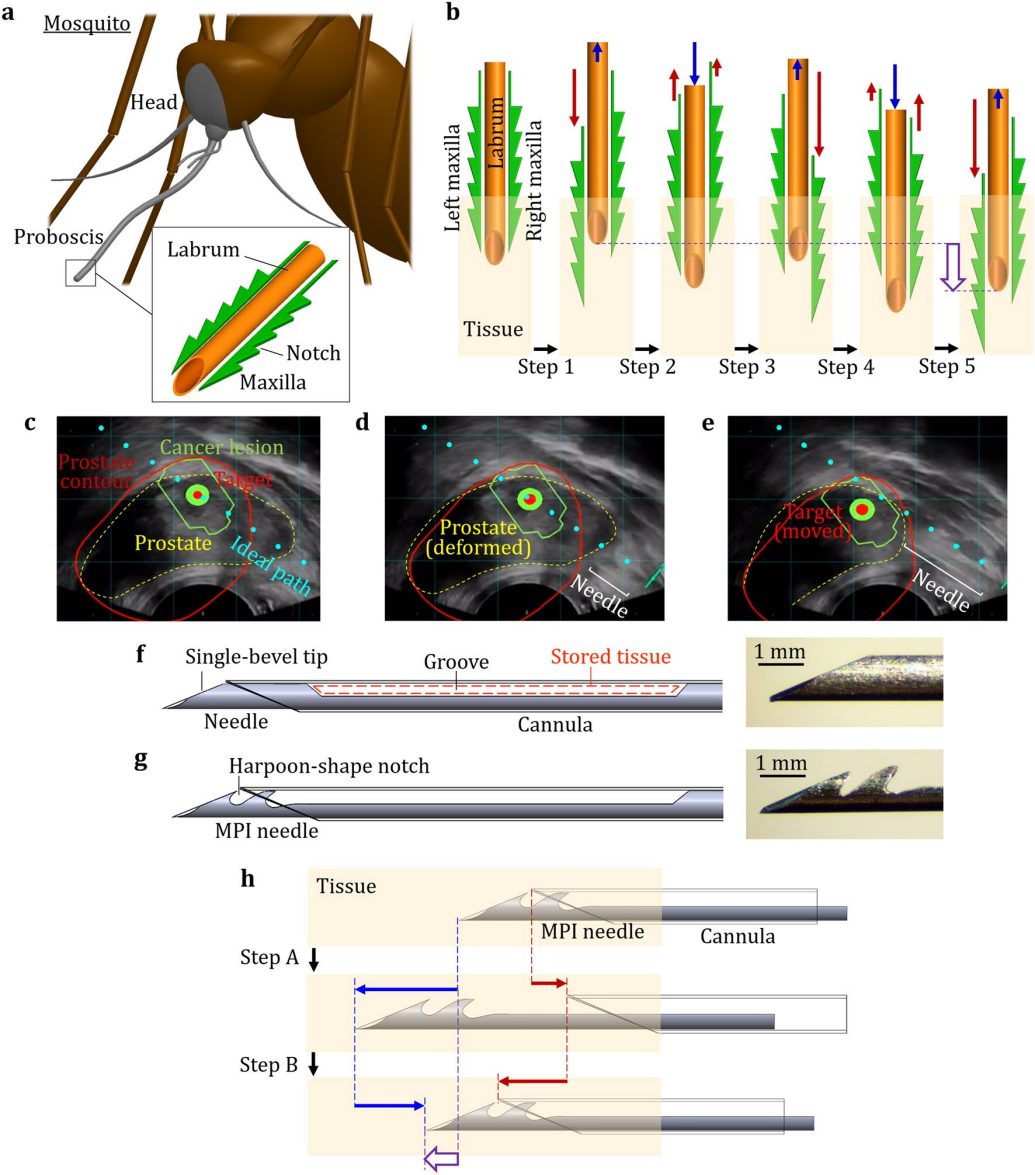
(**a**) Mosquito proboscis has a hollow labrum and two harpoon-shape notched-maxillae. (**b**) The incremental motion during the proboscis insertion reduces insertion force and surrounding tissue deformation. (**c**) The fused magnetic resonance-ultrasound image with a pre-identified cancer lesion allows the targeted sampling of specific lesions in prostate needle biopsy. (**d**) The currently available needle delivery system deforms and moves the prostate. (**e**) The deformation and displacement of the prostate and surrounding tissue move the target location away from the predicted path just prior to biopsy. (**f**) The current trucut biopsy needle comprises an outer hollow cannula and an inner solid needle. (**g**) The mosquito-proboscis inspired (MPI) needle comprises an outer hollow cannula and a needle with two harpoon-shape notches at the tip. (**h**) The needle-cannula incremental motion for vibratory insertion aims to reduce tissue deformation and displacement during MPI insertion.

Mosquito proboscis is an ideal needle device which minimizes the deformation and displacement of surrounding tissue during insertion for accurate guidance to targeted vessels. The proboscis has a hollow labrum (about 25 μm wide) and two maxillae (about 15 μm wide) with harpoon-shape notches on the side^1^ as shown in Fig. 1a. During insertion, the proboscis advances incrementally with vibratory relative displacements of the labrum and maxillae for reciprocating tissue penetration^2,3^. A study proposed the mechanism of proboscis insertion^1^ as illustrated in Fig. 1b. In Step 1, the left maxilla moves forward into the tissue while the labrum retracts a shorter distance in the opposite direction. In Step 2, the labrum moves forward while the maxillae retract utilizing their notches to anchor the surrounding tissue. The forward motion of the right maxilla and backward motion of the labrum in Step 3 mirror the motions in Step 1. In Step 4, movements of both maxillae and the labrum in Step 2 are repeated. After moving the left maxilla forward and the labrum backward in Step 5, the relative positions of the maxillae and labrum are the same as in Step 1. At this state, the proboscis has moved forward by a distance marked by the wide arrow in Fig. 1b. By repeating the above steps, the proboscis incrementally advances with vibratory motions. The vibratory reciprocating motions of mosquito proboscis have been found to reduce insertion force and resultant tissue deformation^3,4^. The harpoon-shape notches of the maxillae may further provide critical support and anchoring to reduce tissue displacement during the proboscis insertion^1,5^.

Tissue deformation and displacement during needle insertion are major issues for accurate needle biopsy in clinical procedures^6–9^. Advances in medical imaging, specifically multi-parametric magnetic resonance imagining (MRI), have enabled the early-stage identification of suspicious-cancerous lesions^10–13^. Needle biopsy is used as a minimally-invasive procedure to sample targeted lesions for pathological analysis. Precise needle deployment to the targeted site is essential for accurate cancer diagnosis and treatment^14,15^. Ultrasound is commonly used to guide the needle in clinical biopsy procedures. However, in certain clinical scenarios, ultrasound cannot reliably identify cancerous lesions^16,17^. Co-registered MRI-ultrasound fusion targeted biopsy (MRF-TB) has gained popularity as a technique allowing targeted sampling of specific lesions^18–20^. The co-registration of the pre-biopsy MRI and live ultrasound during the biopsy procedure allows real-time needle navigation to the targeted lesion site. As the use of targeted biopsy techniques expands, heightened accuracy for needle deployment is essential^6–9^ but technically challenging due to tissue deformation and displacement caused by the current needle delivery systems.

Transrectal ultrasound (TRUS) images fused with a targeted lesion identified by MRI from a clinical MRF-TB procedure for prostate needle biopsy are shown in Fig. 1c–e for three needle positions during insertion. In Fig. 1c, the MRF-TB fusion software generates features on the ultrasound image to guide the clinicians to deploy the needle to the lesion and its center (marked as the green contour and a red point, respectively) for biopsy. The red line is the prostate contour resulting from the co-registration of the MRI and live TRUS images. The ultrasound probe compresses the prostate and deforms it to the shape shown as the yellow contour. The blue dots indicate the predicted needle path for clinician to follow to reach the targeted lesion site.

During the procedure, the needle is first inserted through the rectum wall into the prostate tissues at a low speed. Such insertion generates the force which pushes the surrounding tissue and deforms the prostate as shown in Fig. 1d. The needle then touches the prostate to the ready position for biopsy, as shown in Fig. 1e. However, the prostate has been deformed and moved significantly and the target location has been moved away from the predicted path just prior to biopsy. The discrepancy between desired and actual target locations (ranging from mm- to cm-scale) leads to needle mistargeting^21^ and lesion undersampling^6–9^, especially for those small (5–10 mm) while clinically significant cancer tumors^8^. Such sampling errors have the clinical impact such as false-negative mis- or non-diagnostic biopsy^22^, potentially reducing the cancer diagnostic accuracy^8^.

The currently available trucut biopsy needle for MRF-TB is shown in Fig. 1f. The trucut needle has a solid needle (inside) with a single-bevel tip and a groove to store the biopsied tissue cut by the hollow cannula (outside). Prior to biopsy, the needle and cannula are inserted together to the targeted lesion site. This insertion process can significantly deform and move the prostate as shown in Fig. 1d, e. In this study, two key ideas inspired by the mosquito proboscis are:

- Harpoon-shape notches at the needle tip: As shown in Fig. 1g, the mosquito proboscis-inspired (MPI) needle has two harpoon-shape notches at the tip to mimic the maxilla notches (Fig. 1a). Notches can potentially increase the needle sharpness^5^, reduce the friction due to the reduced contact area^23^, and anchor the surrounding soft tissue^1, 5^ to reduce its deformation and displacement during insertion.
- Needle-cannula reciprocating motions for incremental insertion: As shown in Fig. 1h, the MPI needle and cannula are advanced incrementally with relative motions to mimic the insertion motions of maxillae and labrum (Fig. 1b). In Step A, the needle moves forward while the cannula retracts to generate the opposite motion to reduce the deformation of surrounding tissue. In Step B, the cannula moves forward while the needle retracts with the notches anchoring the tissue to reduce its displacement during cannula insertion. After a complete cycle of Steps A and B, the needle and cannula move by a distance marked by the wide arrow in Fig. 1h.

Many invertebrates capable of penetrating solid substrates (especially mosquito and wasp) were also investigated in prior research to develop bio-inspired needles to improve needle insertion performance. Two common biological features were observed, the serrated/notched apparatuses and vibratory reciprocating motions^1–4,24–26^, which enabled easier piercing/penetration. Inspired by these features, an early study demonstrated the feasibility of harpoon-shape notches in a silicon needle tip for insertion into a soft tissue phantom^27^. In experiments of insertion into the tissue-mimicking phantom^1^ and the ex-vivo tissue^28^, the notched needle tips exhibited lower insertion forces when compared to the needle without notches. The insertion of a needle device with multiple incrementally moving needle components without the harpoon-shape notches was studied and showed the independent benefit of incremental motion to reduce the tissue phantom displacement during insertion^29^ and allow deep insertion without buckling^30,31^. For the notched needle with incremental insertion motion, the puncture^23^ and insertion forces^32^ into the tissue phantom were reduced by approximately 40% and 70%, respectively, in comparison to that of a notched needle without incremental motion. The goal of this study is to utilize the above findings to create the MPI insertion (Fig. 1g, h) with potential applications for the existing needle biopsy system and evaluate tissue deformation and displacement during MPI insertion.

In this paper, particle-embedded optically transparent tissue-mimicking phantoms are utilized to experimentally quantify the deformation and displacement of the phantom induced by the needle insertion. The local tissue deformation and global organ displacement (both based on imaging tracking of particles embedded in the phantoms) and needle insertion forces are measured to quantify the effect of MPI needle insertion with incremental motion.

## Results

### Needle insertion motion and force on the tissue

**Figure 2:**
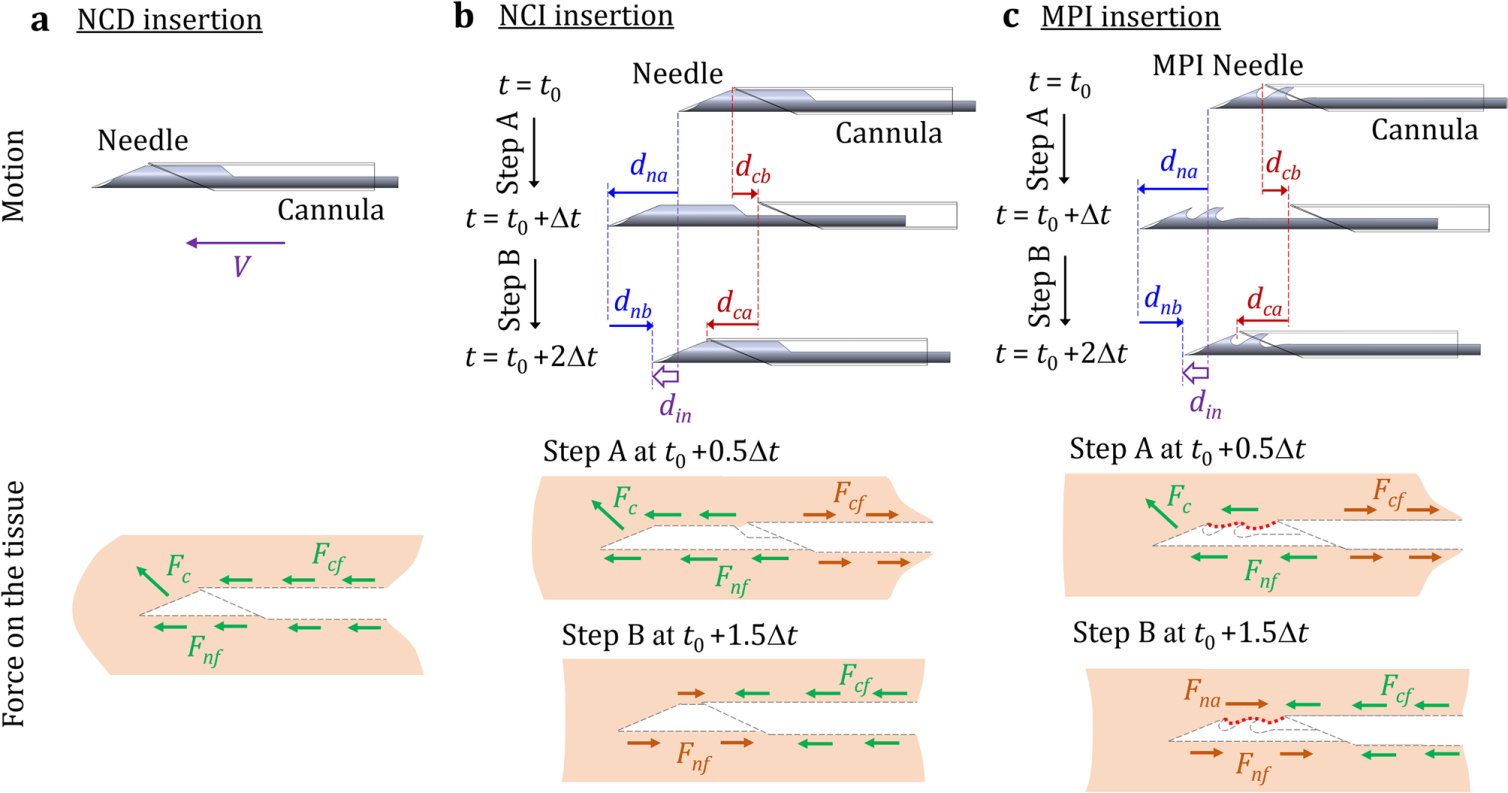
(**a**) NCD insertion (the current needle delivery process): The needle and cannula are inserted together and generate forces on the tissue which deform and push the tissue forward during insertion. (**b**) NCI insertion: the needle (without notches) and cannula are incrementally inserted with the opposite motions to reduce the deformation and displacement of surrounding tissue during insertion. (**c**) MPI insertion: the MPI needle (with notches) and cannula are incrementally inserted with notches anchoring the tissue during insertion to further reduce the overall tissue displacement.

The tissue deformation and displacement of three needle insertion motions, as shown in Fig. 2 and Supplementary Video S1 online (motions at 0.24 playback speed), are investigated and compared. The first is needle-cannula direct (NCD) insertion (Fig. 2a) which is the current needle delivery process to the targeted lesion site prior to biopsy. In NCD insertion, the needle and cannula are inserted together at a constant speed *V*. Three forces, the needle compression force *F*_*c*_, needle friction force *F*_*nf*_, and cannula friction force *F*_*cf*_, exert on the tissue during insertion. The *F*_*c*_ at the needle tip contacts and compresses the tissue. The *F*_*nf*_ and *F*_*cf*_ have the same directions as that of the horizontal component of *F*_*c*_, and together generate significant displacement of surrounding soft tissue and decreasing the needle targeting accuracy of insertion (Fig. 1e).

The needle-cannula incremental (NCI) insertion, as shown in Fig. 2b, is the second motion. The needle (without the harpoon-shape notches at the tip) and cannula advance incrementally with the relative vibratory motions in NCI insertion. From *t*_0_ to *t*_0_ + Δ*t* (Step A), the needle moves forward by *d*_*na*_ while the cannula moves backward by *d*_*cb*_. Such opposite motions of needle and cannula generate the *F*_*cf*_ against *F*_*c*_ and *F*_*nf*_, reducing the surrounding tissue deformation. In Step B (from *t*_0_ + Δ*t* to *t*_0_ + 2Δ*t*), the cannula moves forward by *d*_*ca*_ while the needle moves backward by *d*_*nb*_. The *F*_*cf*_ and *F*_*nf*_ are also opposite to each other to reduce the tissue displacement. The needle and cannula can advance by a distance *d*_*in*_ (= *d*_*na*_ − *d*_*nb*_ = *d*_*ca*_ − *d*_*cb*_) after a complete incremental motion cycle of Steps A and B. In this study, the Δ*t* and *d*_*in*_ were designed to make the average insertion speed equal to *V* in NCD insertion to evaluate the benefit of NCI motion under the same *V*.

The mosquito proboscis-inspired (MPI) insertion, as shown in Fig. 2c, is the third motion. The MPI needle with harpoon-shape notches at the tip and cannula advance incrementally using the same needle-cannula motions as in NCI insertion. The harpoon-shape notches at the MPI needle tip anchor the surrounding tissue during insertion. With the same *d*_*na*_, *d*_*nb*_, *d*_*ca*_, and *d*_*cb*_ as in NCI insertion, the *F*_*nf*_ in Step A will be lower due to reduced contact area (as marked by the red dotted line in Fig. 2c). In Step B, MPI needle anchors the surrounding soft tissue by notches, generates an anchoring force *F*_*na*_ in addition to the *F*_*nf*_ to balance the *F*_*cf*_, and further reduces the overall tissue displacement during insertion.

### Optical measurements of local tissue deformation and global prostate displacement

**Figure 3:**
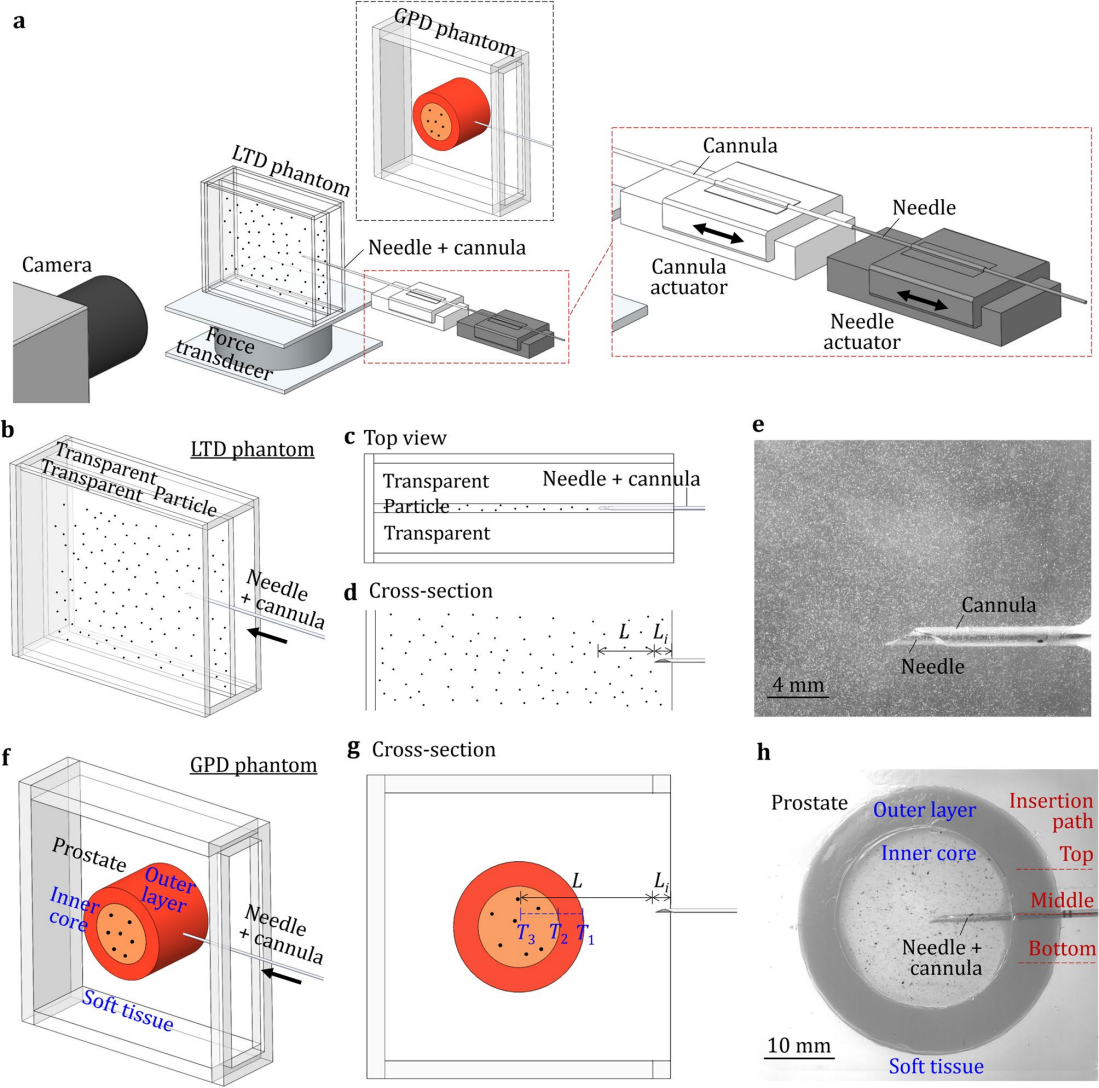
Optical measurements of local tissue deformation and global prostate displacement during needle insertion. (**a**) The experimental setup for needle insertion and optical measurement. (**b**) The LTD phantom, needle, and cannula. (**c**) Top view of the LTD phantom during needle insertion. (**d**) Needle tip positions at the start and end of insertion into the LTD phantom. (**e**) An image of the needle in the LTD phantom at the end of insertion. (**f**) The GPD phantom, needle, and cannula. (**g**) Needle tip positions at the start and end of insertion into the GPD phantom. (**e**) An image of the needle in the GPD phantom at the end of insertion.

This study established the optical measurement methods to quantify tissue deformation and displacement during NCD, NCI, and MPI insertion motions for prostate biopsy (as an example to study organ displacement). The measurement setup is shown in Fig. 3a. The needle and cannula were advanced by the actuators to generate the NCD, NCI, and MPI insertion motions. The transparent polyvinyl chloride (PVC) tissue-mimicking phantom materials with the stiffness and needle insertion properties similar to those of prostate and surrounding muscle tissues^33,34^ are commonly used as the surrogate of soft tissue in needle insertion experimental studies to observe and quantify needle–tissue interaction^21,33,35^. Particles can be embedded in the phantom to further visualize and quantify the deformation and displacement of tissue-mimicking phantoms at the local and global scales^29,36,37^.

Two particle-embedded phantoms, namely the local tissue deformation (LTD) and global prostate displacement (GPD) phantoms, were studied. The LTD phantom had a sandwich-like configuration with a thin particle-embedded PVC layer between two transparent PVC, as shown in Fig. 3a, b. This unique design allowed the camera to easily focus on the particles on/near the needle insertion plane to capture large tissue deformation around the needle without the need for additional illumination. All three layers of PVC had the same elastic modulus and needle insertion properties similar to those of the prostate^38,39^. The needle and cannula were inserted into the particle-embedded PVC layer, as shown in the top view in Fig. 3c. Figure 3d shows the cross-section of the LTD phantom and the needle tip starting and ending positions of the insertion. The fine aluminum oxide powder was used in the LTD phantom, as shown in Fig. 3e, which enabled suitable particle size for visualization and measurement of local tissue deformation near the needle and cannula during insertion, and provided sufficient resolution for digital image correlation (DIC) analysis to track particle displacements^29^. Three insertions were performed for each motion (NCD, NCI, and MPI) at different locations of the phantom to observe the displacement trends.

The GPD phantom, as shown in Fig. 3a, f, was utilized to measure the prostate’s overall displacement during the needle insertion. The GPD phantom modeled the prostate with the soft outer layer, the hard inner core, and the surrounding soft tissues to mimic the in-vivo inhomogeneous prostate tissues^34^. The inner core was embedded with coarse particles to visualize the prostate displacement during needle insertion. Figure 3g shows the cross-section of the GPD phantom and the needle tip position at the start and end of an insertion test. The fine ground pepper powder was used in the GPD phantom, as shown in Fig. 3h, which has a suitable particle size to allow the camera to capture the entire prostate displacement at a larger camera view compared to the LTD experiment. The average displacement of tracking points on particles in the inner core was quantified to represent the global prostate displacement in needle insertion. Each of the three insertion motions (NCD, NCI, and MPI) was repeated in three paths (top, middle, and bottom), as illustrated in Fig. 3h, to evaluate the prostate displacement under different insertion paths (considering the variations of lesion locations in a clinical prostate biopsy) and observe the relative displacement trends.

### Results of local tissue deformation

**Figure 4:**
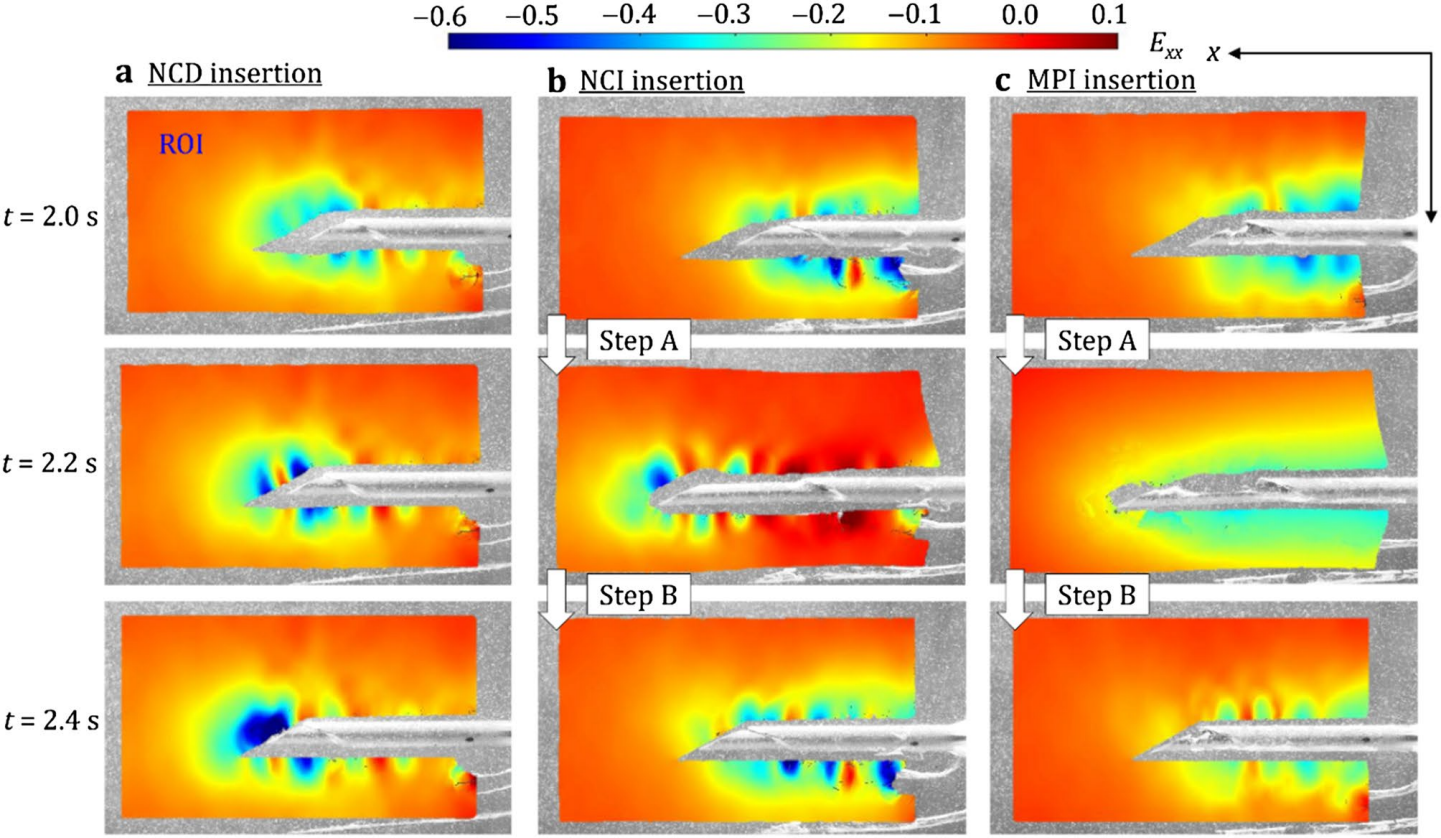
Tissue strain *E*_*xx*_ during three insertion motions at *t* = 2.0, 2.2, and 2.4 s (scale: cannula diameter of 1.27 mm). (**a**) NCD insertion: the direct insertion motion generated the highly concentrated compressive tissue strain in front of the needle which could induce prostate displacement. (**b**) NCI insertion: the opposite motions of the needle and cannula reduced the tissue strain in front of the needle and stabilized the surrounding tissue displacement. (**c**) MPI insertion: the notches at the MPI needle tip provided the critical tissue anchoring to further reduce the overall tissue displacement.

The local deformation of LTD phantom during the needle insertion was quantified as the tissue strain and displacement in DIC (see Supplementary Video S1 online for the DIC animations with the motions at 0.24 playback speed). Figure 4 shows the distributions of tissue strain at the last three time steps (*t* = 2.0, 2.2, and 2.4 s) for NCD, NCI, and MPI insertions. A region of interest (ROI) with the size of 800 × 455 pixels (about 17.2 × 9.6 mm) in the captured image was first defined as the tissue region around the needle and cannula. Inside the ROI, pixels overlapping with the needle and cannula during the insertion were removed to avoid DIC tracking errors. The strain on the *x*-axis (the needle insertion direction), denoted as *E*_*xx*_, is presented as Eulerian strain to visualize the ROI deformation over time (Eulerian view)^40^ for qualitative comparisons among the three motions.

In NCD insertion (Fig. 4a), the needle and cannula were inserted at a constant speed and compressed the tissue in front of the needle. At *t* = 2.0 s, the *E*_*xx*_ in front of the needle tip was about − 0.2. As needle continued advancing, the compressive *E*_*xx*_ in front of the needle tip was increased to about − 0.5 (with two local concentrated peaks) and − 0.6 at 2.2 and 2.4 s, respectively. Such nonuniform, stick–slip deformation had been reported in the force measurement of needle insertion at a constant speed^35,41^. During the insertion, the tissue was compressed to a threshold level and then the cutting occurred. The high compressive strain and force in front of the needle during the insertion pushed the tissue forward, induced the prostate displacement, and decreased the needle targeting accuracy in biopsy (Fig. 1e). The intermittent cutting and nonuniform deformation created ripples of local concentrated deformation in strain distribution around the needle and cannula surfaces^36^. The *E*_*xx*_ around the cannula remained about − 0.1 to − 0.2 over the entire insertion due to the friction force under the constant insertion speed. Away from the needle, the tissue was generally under the low strain with *E*_*xx*_ of about 0.0.

**Figure 5:**
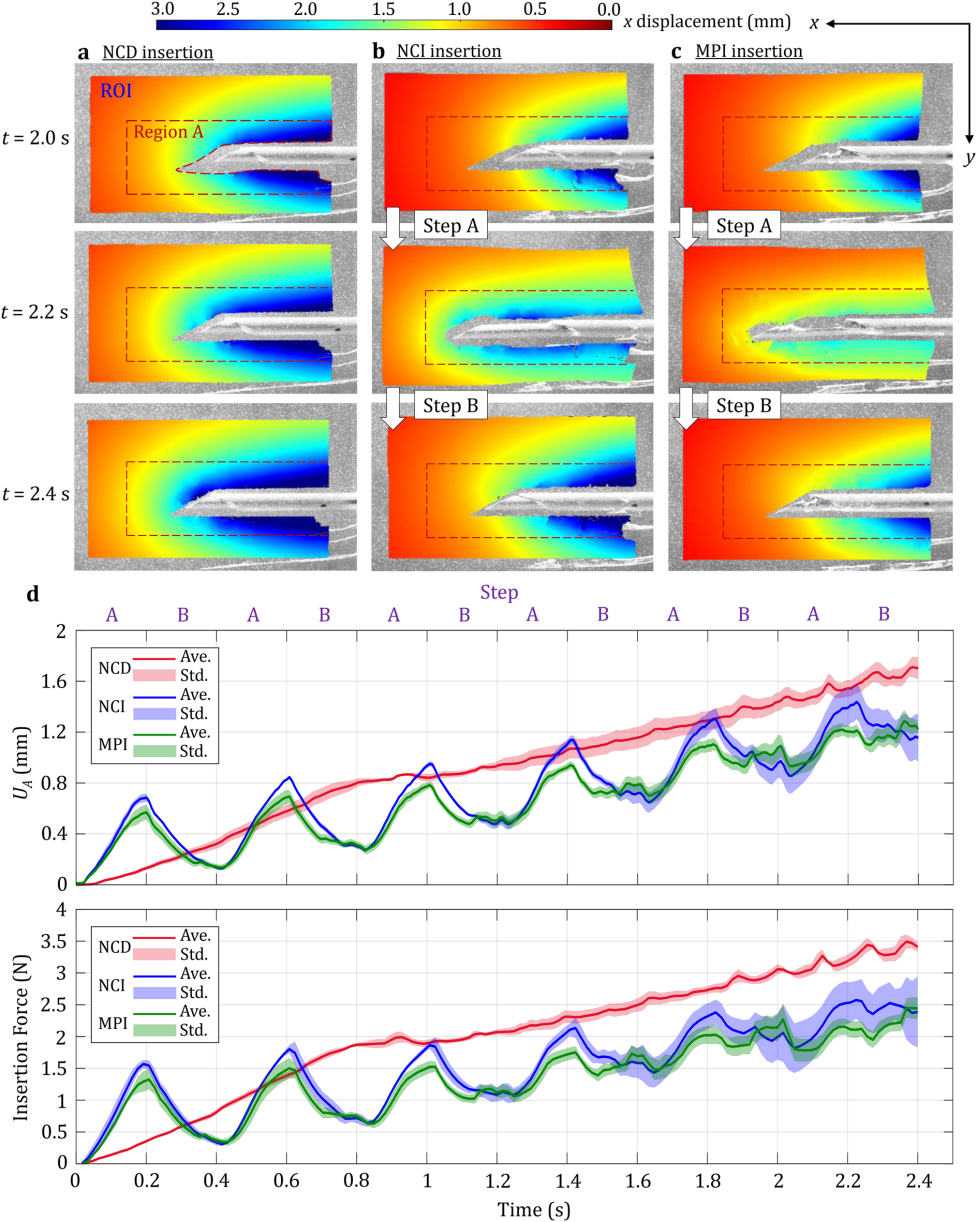
The distribution of tissue displacement at *t* = 2.0, 2.2, and 2.4 s (scale: cannula diameter of 1.27 mm): (**a**) NCD insertion: the direct insertion motion induced the largest displacement of surrounding tissue and force during the insertion, (**b**) NCI insertion: the opposite motions of needle and cannula stabilized and reduced the displacement of surrounding tissue, and (**c**) MPI insertion: notches at the MPI needle tip provided the anchoring to further reduce the overall tissue displacement during insertion. (**d**) The average overall displacement in Region A (*U*_*A*_) and the needle insertion force during NCD, NCI, and MPI insertions over the entire 2.4 s duration. The solid line and the shaded region represent the average value and one standard deviation from the three insertions for each motion, respectively. (Ave. = Average, Std. = Standard deviation).

In NCI insertion (Fig. 4b), at *t* = 2.0 s, the needle and cannula just finished retracting and advancing, respectively. The compressive strain in front of the needle tip was small (*E*_*xx*_ of about − 0.1 to − 0.15) compared to that in NCD insertion. The strain of tissue around the cannula was rippled with the compressive *E*_*xx*_ about − 0.3 to − 0.4, much larger than that in NCD insertion due to cannula’s advancing motion which compressed the tissue. At *t* = 2.2 s, the needle and cannula just finished advancing and retracting, respectively. The needle tip compressed the tissue to *E*_*xx*_ of about − 0.35 to − 0.45. The tissue around the cannula was in tension with *E*_*xx*_ of about 0.1 due to the cannula retraction which pulled and deformed the tissue. Such tissue tension near the cannula could balance the tissue compression around the needle tip, stabilizing the surrounding tissue (to be discussed in Fig. 5). At *t* = 2.4 s, the needle and cannula finished retracting and advancing, respectively, as at *t* = 2.0 s. The distribution of *E*_*xx*_ was also similar to that at *t* = 2.0 s. The balance of compressive and tensile *E*_*xx*_ reduced the overall displacement of the surrounding tissue during insertion (to be discussed in Fig. 5).

In MPI insertion (Fig. 4c), at *t* = 2.0 s, the needle and cannula finished retracting and advancing, respectively, as in NCI insertion. Compared to the *E*_*xx*_ in NCD and NCI insertions, the tissue compressive strain in front of the needle was even smaller with *E*_*xx*_ of about − 0.1 because of the notches at the needle tip. The *E*_*xx*_ around the cannula had fewer ripples (likely due to less tissue displacement) compared to NCI insertion and was reduced to about − 0.2 to − 0.3. At *t* = 2.2 s, as in NCI insertion, the needle and cannula finished advancing and retracting, respectively. The strain in front of the needle was also small with *E*_*xx*_ of about − 0.15 due to the reduced friction force as a result of the notches and reduced contact area at the needle tip. Compared to NCI insertion, the *E*_*xx*_ of about − 0.25 around the cannula was compressive (instead of tensile in NCI) and more uniform. Since the *E*_*xx*_ in front of the needle tip was low due to the reduced friction force, the deformation of tissue was also small. At *t* = 2.4 s, as in NCI insertion, the needle and cannula finished retracting and advancing, respectively. The *E*_*xx*_ of about − 0.1 in front of the needle was the smallest among three insertions. The *E*_*xx*_ around the cannula was also reduced (compared to that of NCI insertion) to about − 0.2 due to notches at the needle tip anchoring the tissue during the retraction. Such anchoring balanced the tissue deformation caused by cannula advancement and needle retraction as well as reducing the overall displacement of the surrounding tissue (to be discussed in Fig. 5).

Figure 5 shows the results of tissue displacement and needle insertion force during NCD, NCI, and MPI insertions. Figure 5a–c are the distribution of tissue displacement (analyzed using the DIC) along the *x*-axis within the ROI at *t* = 2.0, 2.2, and 2.4 s. The displacement is also presented in the Eulerian view to visualize the ROI deformation. In NCD insertion (Fig. 5a), the needle and cannula steadily moved forward and deformed the tissue. At *t* = 2.0 s, the tissue displacement around the cannula was large, about 3 mm, and gradually decayed to about 1.0 mm in front of the needle tip. From *t* = 2.0 to 2.4 s, the tissue displacement in front of the needle increased from 1.0 to 1.5 mm due to the direct insertion motion.

In NCI insertion (Fig. 5b), at *t* = 2.0 s, the tissue displacement was reduced by about 0.5 mm in front of the needle compared to that in NCD insertion, due to the opposite motions of the needle and cannula. At *t* = 2.2 s, the tissue displacement in front of the needle tip and around the cannula was similar at about 1.5 to 2.5 mm. The distribution of tissue displacement was more uniform compared to that in NCD insertion. This was due to the cannula retraction which generated the tissue tension to balance the compression caused by the needle advancement (Fig. 4b) and stabilize the surrounding tissue. At *t* = 2.4 s, the tissue displacement was also much smaller, only about 1 mm in front of the needle tip compared to about 1.5 mm in NCD insertion. This was caused by the needle retraction to reduce the tissue deformation caused by the cannula advancement (Fig. 4b), similar to that at *t* = 2.2 s.

In MPI insertion (Fig. 5c), at *t* = 2.0 s, the distribution of tissue displacement was similar to that in NCI insertion. At *t* = 2.2 s, the tissue displacement in front of the needle tip and around the cannula was further reduced to about 1.0 to 2.0 mm. This was due to the reduced friction force and tissue deformation in front of the needle (Fig. 4c) as a result of the notches at the needle tip. At *t* = 2.4 s, the tissue displacement was about 0.5 mm in front of the needle tip, the smallest in comparison to NCD and NCI insertions. The notches at the needle tip provided the critical tissue anchoring to balance the deformation caused by the cannula during the insertion (Fig. 4c).

Figure 5d shows the overall tissue displacement by averaging the Lagrangian displacement values within Region A with the size of 560 × 250 pixels (about 14.8 × 5.3 mm) to quantify the displacement of the tissue region further surrounding needle and cannula in the ROI (as illustrated by the dash line enclosures in Fig. 5 and denoted as *U*_*A*_) vs. time during the entire 2.4 s insertion. Results of *U*_*A*_ for three insertions under each motion were then averaged as the solid lines shown in Fig. 5d. The shaded regions represent one standard deviation for each motion. Figure 5d also shows the measured needle insertion forces vs. time. The insertion force presented here is the sum of needle tip force (cutting and contact forces), tissue pressure (compression force), and friction force (increasing with insertion length)^21^. For NCD insertion, the *U*_*A*_ and insertion force both kept increasing due to the continuous tissue compression by the direct insertion motion (Fig. 5a). Some small fluctuations on both *U*_*A*_ and insertion force were observed near the end of the insertion (from 1.8 to 2.4 s). This was potentially due to the stick–slip tissue deformation caused by the friction on the cannula surface during insertion, where the slip deformation phenomenon increased with the cannula insertion length, as shown in Fig. 4a and Supplementary Video S1 online.

For NCI insertion, the *U*_*A*_ and insertion force both oscillated as a result of the incremental insertion where the needle advanced more and retracted less to create an incremental advancement. At the beginning of the insertion, compared to NCD insertion, the *U*_*A*_ was larger due to the longer travel in Step A (Fig. 5b), while much smaller after completing Step B due to the needle retraction. When the needle and cannula were further inserted, the *U*_*A*_ was significantly reduced with a smaller increase in *U*_*A*_ after each incremental motion cycle (Steps A and B) compared to NCD insertion. The insertion force also reflected the same trend. This highlights the benefits of NCI insertion with the opposite needle-cannula motions to balance and stabilize the local tissue displacement (Figs. 4b and 5b). It was observed that the magnitudes of oscillations for both *U*_*A*_ and force decreased while the standard deviation increased near the end of the insertion. Similar to NCD insertion, this was likely due to the stick–slip tissue deformation caused by the friction which introduced the variations in displacement results.

For MPI insertion, compared to NCI insertion, the oscillation patterns in *U*_*A*_ and insertion force were also observed while the magnitudes of oscillations in *U*_*A*_ were further reduced due to the notches on the needle tip. The notches not only reduced the friction during the needle advancement but also provided critical anchoring during retraction, further stabilizing and reducing the tissue displacement during the opposite needle-cannula motions (Figs. 4c and 5c). However, the *U*_*A*_ of MPI insertion became similar to that of NCI insertion near the end of the insertion. This was because: (1) the cannula and its motion in NCI and MPI insertions were identical and (2) the friction on the cannula surface dominated the tissue deformation as the cannula was further inserted, making the effect of notches indistinguishable at the local scale.

### Results of global prostate displacement

**Figure 6:**
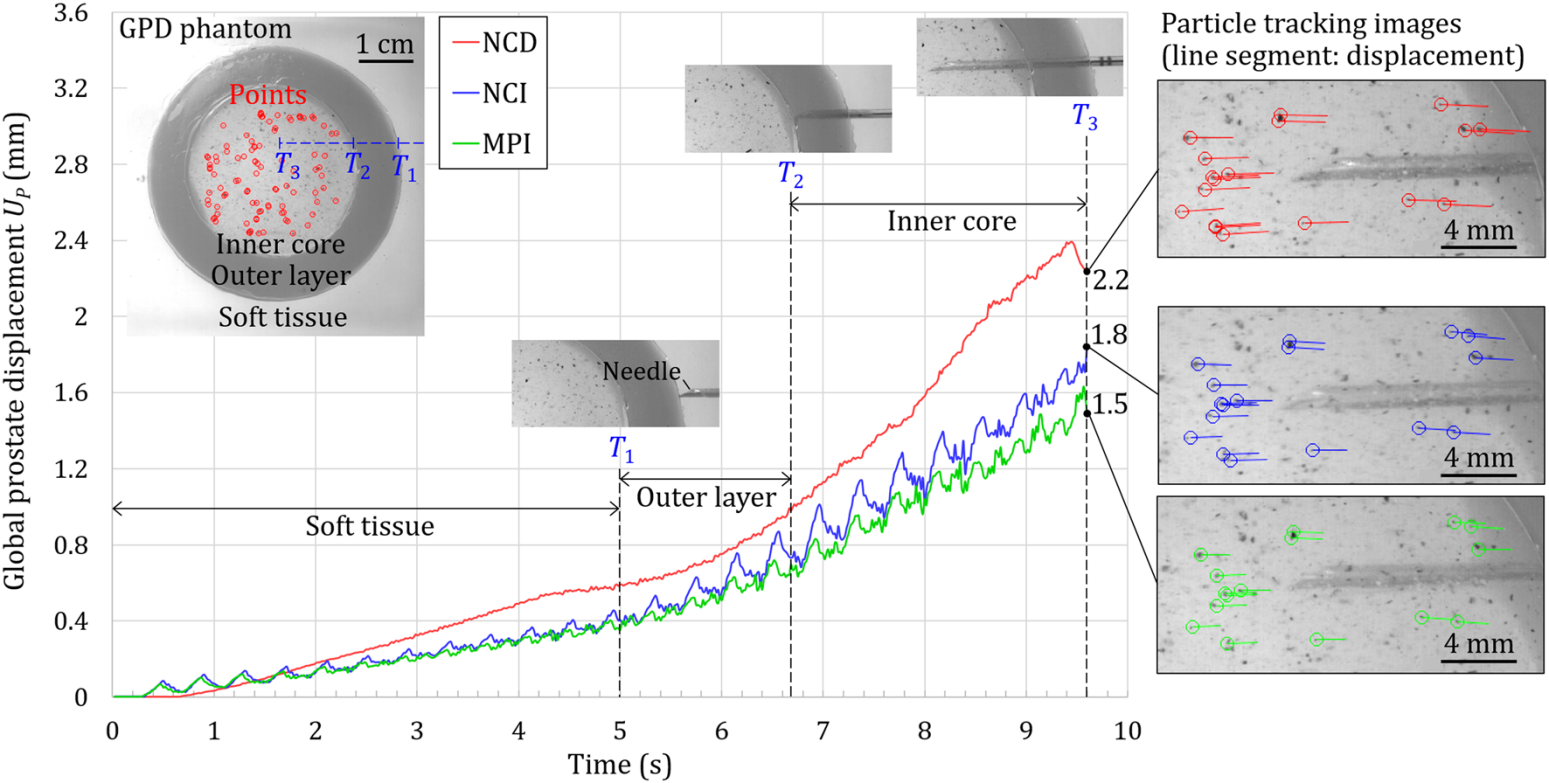
The global prostate displacement *U*_*P*_ vs. time during NCD, NCI, and MPI insertions in the top path. The NCD insertion continued increasing the *U*_*P*_ until the slight drop near the end of the insertion due to the direct insertion motion. The NCI insertion reduced the *U*_*P*_ by the opposite needle-cannula motions. The MPI insertion had the smallest *U*_*P*_ since the notches at the tip provided the reduced friction and tissue anchoring. Images in the particle tracking analysis at the end of each insertion were also shown with line segments representing the tracking point displacement.

The GPD phantom, as shown in Fig. 3f–h, was utilized to track and quantify the global prostate displacement during the needle insertion (see Supplementary Video S1 online for the phantom tracking animations). As illustrated by the red circles in the inner core of the GPD phantom in Fig. 6, 100 trackable points on the particles were automatically identified by the tracking algorithm before the needle insertion. During the needle insertion, the imaging processing algorithm continued tracking these 100 points, removed those points which overlapped with the needle during insertion, and calculated the point displacement between the initial and deformed positions. The global prostate displacement, denoted as *U*_*P*_, was defined as the average of tracking point displacements at a specific time. Figure 6 shows the *U*_*P*_ vs. time during the NCD, NCI, and MPI insertions of the top needle path (as illustrated in Fig. 3h). Three timepoints, *T*_1_, *T*_2_, and *T*_3_ (also illustrated in Fig. 3g), were defined as the timepoints when the needle tip reaches the edge of the outer layer, the edge of the inner core, and at the end of the needle travel, respectively, in the NCD insertion. The *U*_*P*_ for NCI and MPI insertions at the same timepoints were extracted for comparisons.

In NCD insertion, the *U*_*P*_ continued increasing with time until the slight drop near the end of the insertion (9.4 s), potentially due to the slipping of tissue on the needle and cannula to cause the change in displacement (similar to the cause of fluctuations observed in *U*_*A*_ in Fig. 5d). The *U*_*p*_ is equal to 0.6, 1.0, to 2.2 mm at *T*_1_, *T*_2_, and *T*_3_, respectively. This increase of *U*_*P*_ matched to the trend of steadily increasing *U*_*A*_ in Fig. 5a.

In NCI insertion, the *U*_*P*_ was 0.4, 0.7, to 1.8 mm (lower than the 0.6, 1.0, and 2.2 mm in NCD insertion) at *T*_1_, *T*_2_, and *T*_3_, respectively, due to the opposite needle-cannula motions during the insertion. Such opposite motions also caused the oscillations of *U*_*P*_ (also observed in *U*_*A*_ in Fig. 5b). At the beginning of the insertion (less than 1.3 s), the *U*_*P*_ was larger than that of NCD insertion due to the unsteady tissue deformation. After 1.3 s, the opposite needle-cannula motions reduced the *U*_*P*_ in comparison to that in NCD insertion.

In MPI insertion, the *U*_*P*_ was further reduced compared to that of NCI insertion as a result of reduced friction and tissue anchoring enabled by the notches at the needle, which reduced the tissue displacement in front of the needle (Fig. 5c). The *U*_*P*_ of 1.5 mm at *T*_3_ (vs. 2.2 and 1.8 mm in NCD and NCI insertions, respectively) was the smallest. The notches and tissue anchoring also reduced the oscillation amplitude of *U*_*P*_ during the MPI insertion.

**Figure 7:**
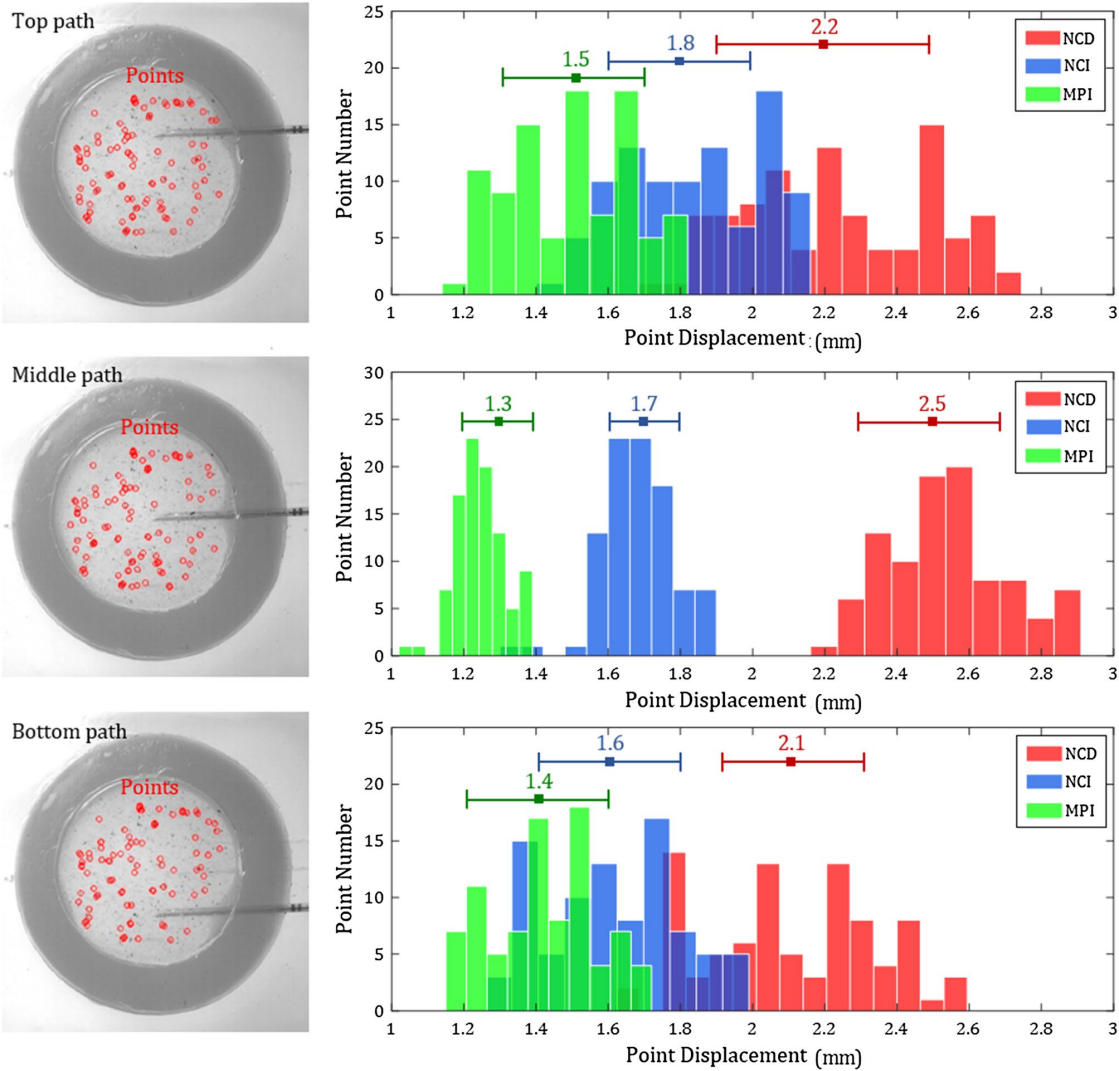
The histogram of tracking point displacements at the end of the NCD, NCI, and MPI insertions in the top, middle, bottom needle paths. The average (*U*_*P*_) and standard deviation of the tracking point displacement are shown at the top of the histogram. For all three insertion paths, the NCD insertion had the largest *U*_*P*_, the NCI insertion had the reduced *U*_*P*_ compared to NCD insertion, and the MPI insertion had the smallest *U*_*P*_.

Figure 7 summarizes the histogram of tracking point displacements at the end of the NCD, NCI, and MPI needle insertions with the top, middle, bottom paths (illustrated in Fig. 3h). The average and standard deviation of the tracking point displacements from the three insertions of each motion are presented. The average of tracking point displacements is equal to *U*_*P*_. The NCD direct insertion motion had the largest *U*_*P*_ of 2.2, 2.5, and 2.1 mm in the top, middle, and bottom paths, respectively. The NCI insertion (with the opposite needle-cannula motion) reduced the *U*_*P*_ to 1.8, 1.7, and 1.6 mm in the top, middle, and bottom paths, respectively. With the notches at the needle tip, the MPI insertion had the smallest *U*_*P*_ of 1.5, 1.3, and 1.4 mm in the top, middle, and bottom paths, respectively. In summary, similar trends of *U*_*P*_ for the three motions were observed in all insertion paths. The differences in *U*_*P*_ along the top and bottom paths were potentially due to the experimental variations caused by nonuniform tissue deformation (stick–slip deformation) and uneven particle distribution.

## Discussion

This study created MPI insertion using the existing biopsy needle and investigated the local tissue deformation and global prostate displacement, quantified as *U*_*A*_ and *U*_*P*_, respectively, during NCD, NCI, and MPI insertions with the same average insertion speed to preliminarily assess the benefits of needle-cannula motions and notches at the needle tip. The opposite needle-cannula motions (mimicking mosquito proboscis’s insertion^1–3^) in NCI and MPI insertions reduced the *U*_*A*_ and *U*_*P*_ compared to those of NCD insertion. The incremental advancements of needle and cannula generated vibratory insertion similar to that of a mosquito proboscis for easier penetration into soft tissue^4^. The cannula/needle retraction further created the opposite force to reduce the tissue deformation caused by the needle/cannula advancement during the incremental insertion. The tissue and organ displacement could also be stabilized and reduced^4,29^. The harpoon-shape notches at the needle tip (mimicking the mosquito’s proboscis notches^1,5^) in MPI insertion resulted in the much reduced *U*_*A*_ and *U*_*P*_ among three insertions. During the needle advancement, the notches reduced the contact area (compared to that of the needle without the notches) and resulted in a low friction force^23^ which reduced the tissue deformation (compared to that of NCI insertion). When the needle retracted, the notches were able to anchor the tissue during the opposite needle-cannula motion^1,5^ to further reduce the overall displacements of the surrounding tissue and prostate. The NCD insertion generated large *U*_*A*_ and *U*_*P*_ due to the direct insertion motion which significantly deformed the surrounding tissue (at the local scale) and pushed the prostate forward (at the global scale). Such deformation and displacement could cause the tissue sampling errors shown in Fig. 1d, e and decrease the accuracy of cancer diagnostic in needle biopsy^6–9^.

While this study provided proof of concept for MPI insertion, a systematic study on MPI parameters will be required to further establish the guideline of parameter selection. The notch shape, including notch width and angle, will be studied to identify an optimal notch geometry minimizing insertion force and maximizing tissue anchoring. The motion profile, including the overall insertion speed and individual velocity (speed and movement) of needle and cannula, will be explored to elucidate the effect of various insertion motions on tissue deformation. On the other hand, there is a possibility of increased tissue damage due to the notches of the MPI needle. Insertion experiments using ex-vivo tissue will be needed to examine if MPI insertion will cause clinically significant collateral damage. Implementation of the MPI features into the existing hand-held needle biopsy device and the corresponding biopsy performance will also be investigated to produce an MPI biopsy system suitable for clinical application.

In summary, this study demonstrated the potential ability of MPI needle insertion to reduce tissue deformation and organ displacement in a clinical biopsy procedure. Findings presented here also provide insights of needle–tissue interaction during needle insertion. This can help to guide researchers to develop new technology to improve needle deployment accuracy in biopsy as well as other clinical procedures requiring accurate needle guidance.

## Methods

### Needle preparation and fabrication

The needles for NCD and NCI insertions were the single-bevel needle of a commercially available prostate needle biopsy device (Pro-Mag Ultra Biopsy Needle by Argon Medical Devices, Frisco, Texas, USA). This needle has a diameter of 1 mm (18-gauge) and the bevel angle of 23.5°, as shown in Fig. 1f. The MPI needle was fabricated by modifying such single-bevel needle tip with two harpoon-shape notches as shown in Fig. 1g. A thin, 0.17 mm thick diamond grinding wheel was used to create an exploratory notch geometry of about 0.4 mm in width and 0.5 mm in depth between the single-bevel needle tip and groove (Fig. 1f). The orientation of the notches was parallel to the needle tip bevel face (with 23.5° bevel angle) to anchor the surrounding tissue during MPI insertion (Fig. 2c). This geometry was chosen to preliminarily evaluate the effect of the notch on tissue deformation (reduced friction and tissue anchoring, as shown in Figs. 4c and 5c). Furthermore, these notches were applied onto the inner needle instead of the outer cannula (unlike the mosquito proboscis with the serrated maxillae outside as shown in Fig. 1a), aiming to mitigate the risk of tissue collateral damage during MPI insertion while maintaining the effect of tissue anchoring.

### Fabrication of tissue-mimicking phantoms

Two tissue-mimicking phantoms, LTD and GPD phantoms (Fig. 3), were fabricated to measure the local tissue deformation and global prostate displacement, respectively. In this study, the ratio of PVC polymer solution (liquid plastic by M-F Manufacturing, Fort Worth, Texas, USA), softener (plastic softener by M-F Manufacturing, Fort Worth, Texas, USA), and the mineral oil (white mineral oil by W.S. Dodge Oil, Maywood, California, USA) were adjusted to match the stiffness and needle insertion properties to those of prostate tissues^33,34^. Particles were embedded in both transparent phantoms with different particle sizes chosen to visualize the displacement of phantom material during the needle insertion at the local and global scales^29,36,37^.

The LTD phantom had a thin particle-embedded PVC layer between two transparent PVC layers as shown in Fig. 3b. All three layers had the same material properties with the elastic modulus *E* of 21.3 kPa to mimic the healthy prostate tissue^38,39^. The particle-embedded PVC layer contained the fine aluminum oxide powder with 240 mesh size suitable for imaging tracking to quantify the tissue deformation at the local scale (tissue surrounding the needle)^29^. In fabrication, the heated and transparent PVC liquid mixture was poured into a phantom holder (100 mm in length, 80 mm in width and 30 mm in height) to form the first transparent PVC layer with about 14 mm in height. Before this layer was completely cured, the same PVC mixture (as the transparent PVC) blended with the aluminum oxide powder was poured onto the top of the first layer to form a 2 mm thick particle-embedded PVC layer. Finally, the transparent PVC mixture was poured into the holder to form another 14 mm thick transparent PVC layer. This unique sandwich-like phantom design enabled the camera to be able to focus on particles surrounding the needle (on/near the needle insertion plane as shown in Fig. 3e) to capture large tissue deformation at the contact area between the needle and tissue without the need for additional illumination.

The GPD phantom, as shown in Fig. 3f, had a round-shaped prostate phantom with the outer layer (40 mm in diameter), inner core (25 mm in diameter), and surrounding soft tissue to mimic the in-vivo prostate tissues^34^. The *E* of the inner core, outer layer, and surrounding soft tissue was 41.7, 21.3, and 9.4 kPa, respectively (all within the range measured by elastography ultrasound of a healthy prostate and surrounding tissue^38,39^). As shown in Fig. 3h, the inner core was embedded with the coarse particles (fine ground pepper powder suitable for image tracking at the global scale^36^) for tracking and measuring the entire prostate displacement. In fabrication, the PVC liquid mixture (for *E* = 41.7 kPa) was first blended with about pepper powder and poured into a cylinder mold (inner diameter of 25 mm) placed in the middle of a 100 mm × 100 mm phantom holder (30 mm deep). Next, the PVC mixture (for *E* = 21.3 kPa) was poured into a cylinder mold with a 40 mm inner diameter and concentric to the inner core to mold the outer layer. This outer layer was dyed in red to illustrate different tissue regions. Finally, the PVC mixture (for *E* = 9.4 kPa) filled the rest of the space to mimic the surrounding soft tissue.

### Needle insertion experimental setup

The needle insertion experimental setup is shown in Fig. 3a. The LTD or GPD phantom was fixed on a force transducer (Gamma F/T Sensor by ATI, Apex, North Carolina, USA) to measure the needle insertion force. A high-speed camera (Model 100K by Photron, San Diego, California, USA) with 1,024 × 1,024 pixel resolution was used to record the needle insertion for imaging tracking (to be discussed in the DIC analysis section). The needle and cannula were attached to two linear actuators (Model HLD 60 by Moog Animatics, Mountain View, California, USA), which were programmed using the motor control interface (SmartMotor Interface by Moog Animatics, Mountain View, California, USA) to generate the NCD, NCI, and MPI insertion motions (Fig. 2). The constant needle insertion speed *V* for NCD insertion (Fig. 2a) was 5 mm/s which was chosen to study the effect of motion on tissue deformation during the low-speed insertion in prostate biopsy (the scope of this study) and fell within the common insertion speed range in clinical needle intervention procedures^42^. For NCI and MPI insertions (Fig. 2b, c), the incremental motion parameters were Δ*t* = 0.2 s, *d*_*na*_ = 4 mm, *d*_*nb*_ = 2 mm, *d*_*ca*_ = 3 mm, *d*_*cb*_ = 1 mm, and *d*_*in*_ = 2 mm. These exploratory parameters were chosen as an early investigation to match the same average *V* of 5 mm/s as in NCD insertion while allowing the individual motions of needle and cannula to have distinct effects on tissue deformation (compressing or pulling the tissue).

### Experimental design

Two needle insertion experiments were performed using the LTD and GPD phantoms for measurements of the local tissue deformation and global prostate displacement, respectively. In the LTD phantom experiment, the needle tip was positioned with *L*_*i*_ = 3 mm and then inserted by *L* = 12 mm, as shown in Fig. 3d, to have a close observation of the needle–tissue interaction at the local scale (tissue surrounding the needle and cannula), aiming to visualize the deformation balancing phenomenon caused by the opposite needle-cannula motions. Three insertions for each motion (NCD, NCI, and MPI, total 9 insertions) were performed at different locations of the LTD phantom. The high-speed camera with 125 frames per second (fps) was used to take images of embedded particles during needle insertions. The captured images were analyzed using DIC to track particle displacements and quantify the local tissue deformation during insertion.

In the GPD phantom experiment, the needle tip was positioned with *L*_*i*_ = 3 mm and then inserted by *L* = 48 mm to make the needle penetrate through inhomogeneous prostate tissues including the soft tissue, outer layer, into the inner core^34^, as shown in Fig. 3g. The *P*_1_, *P*_2_, and *P*_3_ were defined as the three needle tip positions at the edge of outer layer, edge of the inner core, and end of the needle insertion, respectively. Each insertion motion (NCD, NCI, and MPI) was repeated three times for the top, middle, and bottom paths, as shown in Fig. 3h, which were selected to evaluate the prostate displacements under different insertion paths to account for the variations of lesion locations in a clinical prostate biopsy procedure, and observe the displacement trends among the three insertion motions. The high-speed camera with 60 fps was used to capture the displacement of the particles embedded in the inner core of the GPD phantom. The global prostate displacement during insertion was measured using the particle tracking algorithm.

### DIC for local tissue deformation measurement

The DIC analysis was applied to measure the tissue deformation (Figs. 4 and 5). The open-source 2D-DIC software (Ncorr^40^) implemented in MATLAB (R2015 by MathWorks, Natick, Massachusetts, USA) was utilized. A ROI with the size of 800 × 455 pixels (about 17.2 × 9.6 mm) in the captured image (1,024 × 1,024 pixels) was defined around the needle and cannula, as shown in Figs. 4 and 5. Inside the ROI, pixels around the needle and cannula were removed to avoid DIC tracking errors due to the severe particle displacements. The backward DIC analysis in Ncorr using the last frame as the reference to back-calculate the tissue deformation during the needle insertion was adopted. This approach allowed the measurement of large deformation near needle–tissue interaction boundaries.

In DIC analysis, the ROI was divided into subsets with a radius of 30 pixels and the spacing of 1 pixel between subsets. The DIC algorithm calculated the deformation of surrounding tissue by searching the neighboring subsets and matching the features (based on the patterns generated by the embedded particles) between two successive frames. Both Eulerian-Almansi and Green-Lagrangian strain analyses were performed in the DIC algorithm. In this study, results in Eulerian description were used to visualize the ROI deformation over time^40^ for qualitative comparisons among the motions (Figs. 4 and 5a–c) while results in Lagrangian description were used to quantify the tissue displacement with respect to the undeformed state (Fig. 5d). The local tissue displacement *U*_*A*_ was calculated by averaging the Lagrangian displacement data of each pixel in Region A with a size of 560 × 250 pixels (about 14.8 × 5.3 mm), as illustrated by the dash line enclosures in Fig. 5, to quantify the displacement of the tissue further surrounding the needle and cannula in the ROI (where large tissue deformation existed).

### Particle tracking for global prostate displacement measurement

The particle tracking analysis was applied to measure the global prostate displacement *U*_*p*_ (Figs. 6 and 7). The Kanade-Lucas-Tomasi (KLT) feature tracking algorithm in MATLAB was applied to measure displacements of tracking points in the GPD phantom (Fig. 3h) during the needle insertion^36^. The top 100 trackable points (as illustrated by red circles in Figs. 6 and 7) were identified using the minimum eigenvalue method. These 100 points were tracked frame by frame during insertion. Tracking points that overlapped with the needle during insertion were removed. The displacements of tracking points between the initial and deformed positions for each frame were calculated. The average displacement of all tracking points without overlapping with the needle and cannula was calculated as *U*_*p*_.

## Supplementary information

Supplementary video

## Data Availability

The experimental and simulation data supporting the findings in this study are available within this article and from the corresponding author upon request.
